# Synergistic impact of pre-sensitization and delayed graft function on allograft rejection in deceased donor kidney transplantation

**DOI:** 10.1038/s41598-021-95327-6

**Published:** 2021-08-09

**Authors:** Hanbi Lee, Yohan Park, Tae Hyun Ban, Sang Heon Song, Seung Hwan Song, Jaeseok Yang, Curie Ahn, Chul Woo Yang, Byung Ha Chung, The Korean Organ Transplantation Registry Study Group, Jin Min Kong, Oh Jung Kwon, Deok Gie Kim, Cheol-Woong Jung, Yeong Hoon Kim, Joong Kyung Kim, Chan-Duck Kim, Ji Won Min, Sung Kwang Park, Yeon Ho Park, Park Jae Berm, Jung Hwan Park, Jong-Won Park, Ho Sik Shin, Hye Eun Yoon, Kang Wook Lee, Dong Ryeol Lee, Dong Won Lee, Sam Yeol Lee, Sang-Ho Lee, Su Hyung Lee, Jung Jun Lee, Lee Jung Pyo, Jeong-Hoon Lee, Jin Seok Jeon, Heungman Jun, Kyunghwan Jeong, Ku Yong Chung, Hong Rae Cho, Ju Man Ki, Dong-Wan Chae, Soo Jin Na Choi, Duck Jong Han, Seungyeup Han, Kyu Ha Huh

**Affiliations:** 1 Division of Nephrology, Department of Internal Medicine, Seoul St. Mary’s Hospital, Seoul, South Korea; 2 Division of Nephrology, Department of Internal Medicine, Eunpyeong St. Mary’s Hospital, Seoul, South Korea; 3 Organ Transplantation Center and Department of Internal Medicine, Pusan National University Hospital, Busan, South Korea; 4 Department of Surgery, Ewha Womans University Medical Center, Seoul, South Korea; 5 Department of Nephrology, Seoul National University Hospital, Seoul, South Korea; 6 Present Address: Division of Nephrology, Department of Internal Medicine, Konyang University Hospital, College of Medicine, Konyang University, Daejeon, Republic of Korea; 7 Department of Nephrology, BHS Hanseo Hospital, Busan, South Korea; 8 Department of Surgery, College of Medicine, Han Yang University, Seoul, South Korea; 9 Department of Surgery, Yonsei University Wonju College of Medicine, Wonju Severance Christian Hospital, Wonju-si, South Korea; 10 Department of Transplantation and Vascular Surgery, Korea University Anam Hospital, Seoul, South Korea; 11 Department of Internal Medicine, Inje University Busan Paik Hospital, Busan, South Korea; 12 Department of Internal Medicine, Bongseng Memorial Hospital, Busan, South Korea; 13 Department of Internal Medicine, School of Medicine, Kyungpook National University Hospital, Daegu, South Korea; 14 Division of Nephrology, Department of Internal Medicine, Bucheon St. Mary’s Hospital, Bucheon, South Korea; 15 Department of Internal Medicine, Chonbuk National University Medical School, Jeonju, South Korea; 16 Department of Surgery, Gil Medical Center, Gachon University College of Medicine, Seongnam, South Korea; 17 Department of Surgery, Samsung Medical Center, Sungkyunkwan University School of Medicine, Seoul, South Korea; 18 Department of Nephrology, Konkuk University School of Medicine, Seoul, South Korea; 19 Department of Nephrology, Yeungnam University Hospital, Daegu, South Korea; 20 Division of Nephrology, Department of Internal Medicine, Kosin University College of Medicine, Busan, South Korea; 21 Department of Internal Medicine, Incheon St. Mary’s Hospital, Incheon, South Korea; 22 Department of Nephrology, Chungnam National University Hospital, Daejeon, South Korea; 23 Division of Nephrology, Department of Internal Medicine, Maryknoll Medical Center, Busan, South Korea; 24 Division of Nephrology, Department of Internal Medicine, Pusan National University School of Medicine, Busan, South Korea; 25 Department of Surgery, Kangdong Sacred Heart Hospital, Hallym University College of Medicine, Chuncheon, South Korea; 26 Department of Nephrology, Kyung Hee University Hospital at Gangdong, Seoul, South Korea; 27 Department of Surgery, Ajou University School of Medicine, Suwon, South Korea; 28 Department of Surgery, CHA Bundang Medical Center, Seongnam, South Korea; 29 Department of Nephrology, SNU Boramae Medical Center, Seoul, South Korea; 30 Department of Surgery, Myongji Hospital, Goyang, South Korea; 31 Department of Internal Medicine, Soonchunhyang University Seoul Hospital, Seoul, South Korea; 32 Department of Surgery, Inje University Ilsan Paik Hospital, Goyang, South Korea; 33 Department of Internal Medicine, Kyung Hee University College of Medicine, Seoul, South Korea; 34 Department of Surgery, Ewha Womans University Mokdong Hospital, Seoul, South Korea; 35 Department of Surgery, Ulsan University Hospital, Ulsan, South Korea; 36 Department of Surgery, Gangnam Severance Hospital, Yonsei University College of Medicine, Seoul, South Korea; 37 Division of Nephrology, Seoul National University Bundang Hospital, Seongnam, South Korea; 38 Department of Surgery, Chonnam National University Medical School, Gwangju, South Korea; 39 Department of Surgery, Asan Medical Center, Seoul, South Korea; 40 Department of Internal Medicine, Keimyung University School of Medicine, Daegu, South Korea; 41 Department of Transplantation Surgery, Severance Hospital, Seoul, South Korea

**Keywords:** Medical research, Nephrology

## Abstract

The aim of this study is to investigate whether or not delayed graft function (DGF) and pre-transplant sensitization have synergistic adverse effects on allograft outcome after deceased donor kidney transplantation (DDKT) using the Korean Organ Transplantation Registry (KOTRY) database, the nationwide prospective cohort. The study included 1359 cases between May 2014 and June 2019. The cases were divided into 4 subgroups according to pre-sensitization and the development of DGF post-transplant [non-pre-sensitized-DGF(−) (n = 1097), non-pre-sensitized-DGF(+) (n = 127), pre-sensitized-DGF(−) (n = 116), and pre-sensitized-DGF(+) (n = 19)]. We compared the incidence of biopsy-proven allograft rejection (BPAR), time-related change in allograft function, allograft or patient survival, and post-transplant complications across 4 subgroups. The incidence of acute antibody-mediated rejection (ABMR) was significantly higher in the pre-sensitized-DGF(+) subgroup than in other 3 subgroups. In addition, multivariable cox regression analysis demonstrated that pre-sensitization combined with DGF is an independent risk factor for the development of acute ABMR (hazard ratio 4.855, 95% confidence interval 1.499–15.727). Moreover, DGF and pre-sensitization showed significant interaction (p-value for interaction = 0.008). Pre-sensitization combined with DGF did not show significant impact on allograft function, and allograft or patient survival. In conclusion, the combination of pre-sensitization and DGF showed significant synergistic interaction on the development of allograft rejection after DDKT.

## Introduction

Delayed graft function (DGF) is a manifestation of acute kidney injury (AKI), which is more prevalent in deceased donor kidney transplantation (DDKT). The definition of DGF varies according to the study; however, it is mostly based on the use of dialysis within 1 week from transplant^1–3^. The mechanism underlying the development of DGF still needs to be unveiled, but it is suggested that post-ischemic acute tubular necrosis resulting from ischemia and reperfusion injury (IRI) developing during deceased donor management or recovery of organs, and calcineurin inhibitor (CNI) toxicity may be the major contributors^4^. The activation of adaptive immune system induced by DGF also increases the risk of allograft rejection.

Meanwhile, it is well known that the presence of preexisting donor-specific anti-human leukocyte antigen antibody (HLA-DSA), so-called “pre-sensitized state” is an important obstacle preventing successful kidney transplantation (KT)^5–9^. In such patients, HLA-DSA can increase the risk of acute or chronic antibody-mediated rejection (ABMR) resulting in worse allograft outcomes^10,11^. In the setting of DDKT, DGF combined with subclinical rejection resulted in far worse allograft outcomes. In addition, the detrimental impact of DGF on allograft was enhanced by the presence of pre-transplant HLA-DSA in DDKT^12^.

Based on the above background, it is possible that DGF in patients with pre-sensitization has a synergistic adverse impact on the allograft outcomes. However, it has yet to be fully investigated and only a single center study is available^12^. In this regard, the aim of this study is to investigate the combined impact of DGF and pre-sensitization on the development of allograft rejection using the well-established nationwide prospective cohort, Korean Organ Transplantation Registry (KOTRY).

## Results

### Baseline clinical and immunological patient characteristics

DGF developed in 10.7% (146/1359) out of the total DDKT recipients. Between pre-sensitized and non-pre-sensitized subgroups, no difference was detected in the frequency of DGF (9.6% vs. 13.0%, *p* = 0.188). Table 1 describes baseline characteristics of the donor and recipients of four subgroups. Baseline estimated glomerular filtration rate (eGFR) was significantly lower in donors of DGF(+) subgroups irrespective of pre-sensitization. Cold ischemic time showed a longer tendency in DGF(+) subgroups irrespective of pre-sensitization without statistical significance. However, donor age, gender, body mass index (BMI), underlying disease including DM or hypertension (HTN) and the proportion of donors after cardiac death (DCD) or donors after brain death (DBD) did not differ significantly across 4 subgroups. In our study, 3 had dual-kidney transplantation from expanded criteria donors, and 6 had en-bloc kidney transplantation from pediatric donors. All allocated in non-pre-sensitized-DGF(−) subgroup. There were no pre-emptive transplantation cases.

**Table 1:** Comparison of clinical and laboratory parameters among the 4 subgroups according to DGF and pre-sensitization status.

	Non-pre-sensitized (n = 1224)		Pre-sensitized (n = 135)		*p*-value
	DGF(−) (n = 1097)	DGF(+) (n = 127)	DGF(−) (n = 116)	DGF(+) (n = 19)	
**Donors**					
Age (years)	47.6 ± 14.9	48.7 ± 14.8	49.2 ± 13.6	47.1 ± 13.0	0.615
Male (n, %)	772 (70.4%)	92 (72.4%)	83 (71.6%)	10 (52.6%)	0.361
BMI (kg/m^2^)	23.2 ± 3.7	23.4 ± 3.8	22.9 ± 3.6	23.4 ± 4.9	0.743
HTN (n, %)	260 (25.3%)	36 (29.0%)	21 (18.9%)	3 (17.6%)	0.284
DM (n, %)	120 (11.6%)	13 (10.4%)	12 (10.8%)	1 (5.6%)	0.852
DBD (n, %)	1056 (96.3%)	123 (96.9%)	112 (96.6%)	19 (100.0%)	0.838
DCD (n, %)	41 (3.7%)	4 (3.1%)	4 (3.4%)	0 (0.0%)	0.838
eGFR (CKD-EPI) (ml/min/1.73 m^2^)	77.5 (43.3–107.1)	32.6 (20.6–63.9)*	72.2 (45.9–103.8)^†^	47.8 (25.5–103.0)	< 0.001
Cold ischemic time (min)	290.3 ± 138.0	324.8 ± 135.8	284.4 ± 124.7	322.9 ± 146.0	0.061
KDPI (%)	66.0 (44.0–84.0)	71.0 (51.0–89.0)	64.0 (51.0–81.0)	67.0 (48.0–82.0)	0.221
**Recipients**					
Age (years)	51.3 ± 10.6	52.7 ± 11.1	51.1 ± 9.8	50.1 ± 12.1	0.502
Male (n, %)	678 (61.8%)	75 (59.1%)	38 (32.8%)*^,^^†^	8 (42.1%)	< 0.001
BMI (kg/m^2^)	23.0 ± 3.3	22.9 ± 3.3	22.0 ± 2.9*	23.0 ± 2.8	0.022
HTN (n, %)	986 (90.0%)	115 (90.6%)	97 (84.3%)	19 (100.0%)	0.111
DM (n, %)	311 (28.4%)	37 (29.1%)	17 (14.7%)*^,^^†^	4 (21.1%)	0.014
**Dialysis modality (n, %)**					
Hemodialysis	857 (78.1%)	112 (88.2%)*	97 (83.6%)	18 (94.7%)	0.011
Peritoneal dialysis	240 (21.9%)	15 (11.8%)*	19 (16.4%)	1 (5.3%)	0.011
Dialysis duration (months)	84.5 (53.7–113.4)	90.8 (50.2–114.6)	103.7 (68.5–136.0)*^,†^	130.5 (108.6–164.9)*^,†^	< 0.001
Previous KT history (n, %)	92 (8.4%)	14 (11.0%)	31 (26.7%)*†	3 (15.8%)	< 0.001
Mismatch number (n)	3.4 ± 1.8	3.5 ± 1.6	3.8 ± 1.3*	3.7 ± 1.6	0.023
**Induction therapy (n, %)**					
ATG	309 (28.2%)	58 (45.7%)*	69 (59.5%)*^,^^†^	12 (63.2%)*	< 0.001
Basiliximab	803 (73.3%)	84 (66.1%)	59 (50.9%)*^,^^†^	14 (73.7%)	< 0.001
**Main immunosuppressant (n, %)**					
Tacrolimus	1076 (98.1%)	126 (99.2%)	115 (99.1%)	18 (94.7%)	0.436
Cyclosporin	16 (1.5%)	0 (0.0%)	0 (0.0%)	1 (5.3%)	0.115
Sirolimus	8 (0.7%)	3 (2.4%)	1 (0.9%)	1 (5.3%)	0.073
PRA > 50%	325 (29.6%)	39 (30.7%)	95 (81.9%)*^,^^†^	14 (73.7%)*^,^^†^	< 0.001
Follow-up period (months)	38.2 (25.3–50.9)	37.5 (24.9–51.8)	37.8 (24.7–48.0)	36.0 (17.7–44.1)	0.651

Continuous variables are shown as mean ± standard deviation or median with interquartile range. Categorical variables are shown as number (proportions).

*DGF* delayed graft function, *BMI* body mass index, *HTN* hypertension, *DM* diabetes mellitus, *DBD* donor after brain death, *DCD* donor after cardiac death, *eGFR* estimated glomerular filtration, *CKD-EPI* chronic kidney disease-epidemiology collaboration, *KDPI* kidney donor profile index, *KT* kidney transplantation, *ATG* anti-thymocyte globulin, *PRA* panel reactive antibody, *DSA* donor-specific antibody.

*p < 0.05 compared with non-pre-sensitized-DGF(−) subgroup, ^**†**^*p* < 0.05 compared with non-pre-sensitized-DGF(+) subgroup.

Among recipient factors, there was a significantly longer dialysis vintage and also additional number of female patients in both pre-sensitized subgroups than in non-pre-sensitized subgroups. As expected, pre-sensitized subgroups had higher HLA mismatch number. In addition, a previous KT history and the proportion of anti-thymocyte globulin (ATG) use as induction therapy were higher in the pre-sensitized subgroups than in the non-pre-sensitized subgroups. The proportion of DM as the primary renal disease was lower in the pre-sensitized subgroups than in the non-pre-sensitized subgroups. A significantly higher proportion of patients undergoing hemodialysis as the dialysis modality prior to KT were selected from the non-pre-sensitized-DGF(+) subgroup, when compared with the non-pre-sensitized-DGF(−) subgroup. Although the majority of patients received tacrolimus as the main immunosuppressant, more patients in DGF(+) subgroups showed a tendency to take sirolimus compared with DGF(−) subgroups.

### Comparison of overall biopsy-proven allograft rejection (BPAR) and acute ABMR

The median time to BPAR and ABMR from transplant showed no significant difference across 4 subgroups (BPAR, p = 0.357; ABMR, p = 0.318). Despite the incidence of overall BPAR was not significantly different across 4 subgroups, pre-sensitized-DGF(+) subgroup tended to be higher compared with other 3 subgroups. The incidence of acute ABMR was higher in pre-sensitized-DGF(+) subgroup (21.1%, 4/19) than in other 3 subgroups. Totally, acute ABMR occurred in 53 kidney transplantation recipients (KTRs), and of these, 3 had de novo DSA at the time of biopsy. 1 was in non-pre-sensitized-DGF(−) subgroup, 1 in non-pre-sensitized-DGF(+) subgroup, the other in pre-sensitized-DGF(+) subgroup. The incidence of chronic ABMR was higher in pre-sensitized subgroups compared to non-pre-sensitized subgroups. In contrast, acute and chronic T-cell mediated rejection (TCMR) rate showed no statistically significant difference across 4 subgroups (Table 2).

**Table 2:** Comparison of rejection-related outcomes among the 4 subgroups according to DGF and pre-sensitization status.

	Non-pre-sensitized (n = 1224)		Pre-sensitized (n = 135)		*p*-value
	DGF (−) (n = 1097)	DGF (+) (n = 127)	DGF (−) (n = 116)	DGF (+) (n = 19)	
Overall BPAR (n, %)	139 (12.7%)	17 (13.4%)	17 (14.7%)	6 (31.6%)	0.107
Acute ABMR (n, %)	35 (3.2%)	5 (3.9%)	9 (7.8%)*	4 (21.1%)*^,^^†^	< 0.001
Acute TCMR (n, %)	107 (9.8%)	10 (7.9%)	9 (7.8%)	4 (21.1%)	0.284
Chronic active ABMR (n, %)	3 (0.3%)	2 (1.6%)	5 (4.3%)*	1 (5.3%)	< 0.001
Chronic active TCMR (n, %)	10 (0.9%)	3 (2.4%)	1 (0.9%)	1 (5.3%)	0.152
Repeated acute rejection within 1 year (n, %)	41 (3.7%)	3 (2.4%)	5 (4.3%)	2 (10.5%)	0.363

Categorical variables are shown as number (proportions).

*DGF* delayed graft function, *BPAR* biopsy-proven allograft rejection, *ABMR* antibody-mediated rejection, *TCMR* T-cell mediated rejection.

*p < 0.05 compared with non-pre-sensitized-DGF(−) subgroup, ^**†**^*p* < 0.05 compared with non-pre-sensitized-DGF(+) subgroup.

**Figure 1:**
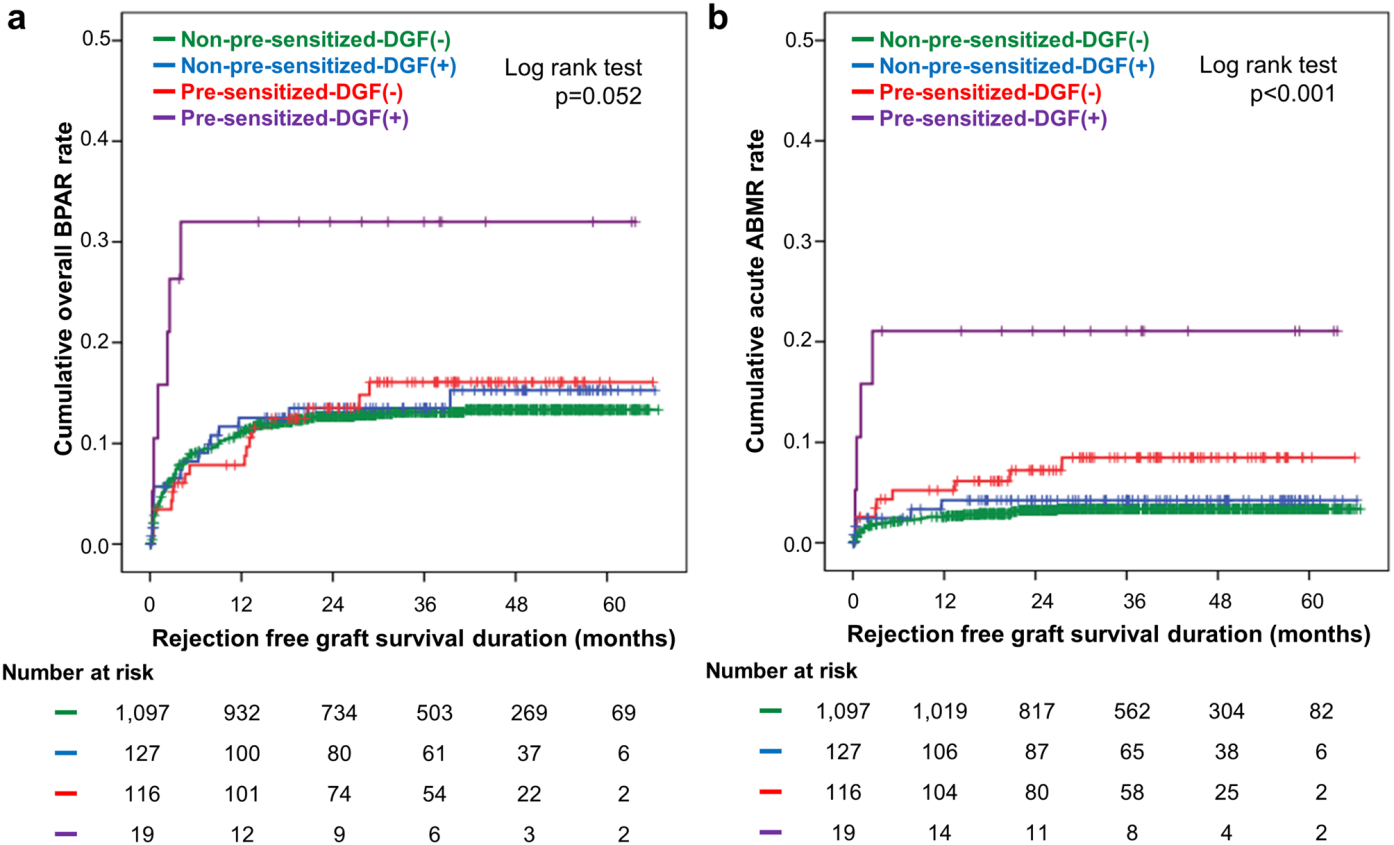
Kaplan–Meier estimates of withdrawal-censored allograft rejection according to DGF and pre-sensitization status. (**a**) Overall BPAR rate, (**b**) acute ABMR rate. The numbers below the figures denote the number of KTRs at risk in each subgroup. *DGF* delayed graft function, *BPAR* biopsy-proven allograft rejection, *ABMR* antibody-mediated rejection, *KTR* kidney transplantation recipient.

Although not significant, the Kaplan–Meier curve showed that cumulative overall BPAR rate had a tendency to be higher in pre-sensitized-DGF(+) subgroup (log rank p = 0.052) (Fig. 1a). Cumulative acute ABMR rate was significantly highest in the pre-sensitized-DGF(+) subgroup [log rank; p < 0.001 vs. non-pre-sensitized-DGF(−), p = 0.004 vs. non-pre-sensitized-DGF(+), p = 0.052 vs. pre-sensitized-DGF(−)] (Fig. 1b).

### Risk factors of overall BPAR and acute ABMR

In cox regression analysis, pre-sensitization and DGF individually were not independent risk factors of overall BPAR [pre-sensitization, hazard ratio (HR) 1.353, 95% confidence interval (CI) 0.874–2.097, p = 0.176; DGF, HR 1.292, 95% CI 0.834–2.001, p = 0.252]. However, when pre-sensitization and DGF were taken together, it became an independent risk factor for overall BPAR (unadjusted HR 2.933, 95% CI 1.299–6.619, p = 0.010, adjusted HR 2.663, 95% CI 1.087–6.525, p = 0.032) (Table 3a).

**Table 3:** Multivariable Cox regression for independent predictors of (a) overall BPAR and (b) acute ABMR.

	Unadjusted HR (95% CI)	*p*-value for interaction	Adjusted HR (95% CI)	*p*-value for interaction
**(a)**				
KDPI	1.006 (1.000–1.013)			
Recipient BMI	1.057 (1.015–1.102)			
Mismatch number	1.136 (1.039–1.242)		1.100 (0.988–1.225)	
Cold ischemic time	0.998 (0.997–1.000)		0.998 (0.997–1.000)	
Non-pre-sensitization	Reference			
Pre-sensitization	1.353 (0.874–2.097)			
DGF (−)	Reference			
DGF ( +)	1.292 (0.834–2.001)			
Pre-sensitization and DGF	2.933 (1.299–6.619)	0.010	2.663 (1.087–6.525)	0.032
**(b)**				
PRA > 50%	1.864 (1.088–3.195)			
Previous KT history	2.291 (1.180–4.450)		3.265 (1.641–6.496)	
Dialysis duration	1.004 (1.000–1.008)			
Non-pre-sensitization	Reference			
Pre-sensitization	2.977 (1.592–5.566)			
DGF(−)	Reference			
DGF(+)	1.787 (0.872–3.660)			
Pre-sensitization and DGF	6.666 (2.404–18.481)	< 0.001	4.855 (1.499–15.727)	0.008

(a) Multivariable regression model was adjusted with parameters showing significant differences in univariable analysis or known to affect overall BPAR. Parameters were as follows: donor factors (cold ischemic time, KDPI), recipient factors (BMI, dialysis duration, previous KT history, mismatch number, PRA > 50%). 1021 (75.1%) recipients were included.

(b) Multivariable regression model was adjusted with parameters showing significant differences in univariable analysis or known to affect acute ABMR. Parameters were as follows: donor factors (cold ischemic time, KDPI), recipient factors (dialysis duration, previous KT history, mismatch number, PRA > 50%). 1,023 (75.3%) recipients were included.

*BPAR* biopsy-proven allograft rejection, *ABMR* antibody-mediated rejection, *HR* hazard ratio, *CI* confidence interval, *KDPI* kidney donor profile index, *BMI* body mass index, *DGF* delayed graft function, *PRA* panel reactive antibody, *KT* kidney transplantation.

In respect of acute ABMR, while DGF alone was not an independent risk factor (HR 1.787, 95% CI 0.872–3.660, p = 0.113), pre-sensitization was associated with a significant HR (HR 2.977, 95% CI 1.592–5.566, p = 0.001). In interaction analysis, the combination of pre-sensitization and DGF had much higher HR (unadjusted HR 6.666, 95% CI 2.404–18.481, p < 0.001, adjusted HR 4.855, 95% CI 1.499–15.727, p = 0.008) (Table 3b).

### Comparison of the change in allograft function and death-censored allograft survival

**Figure 2:**
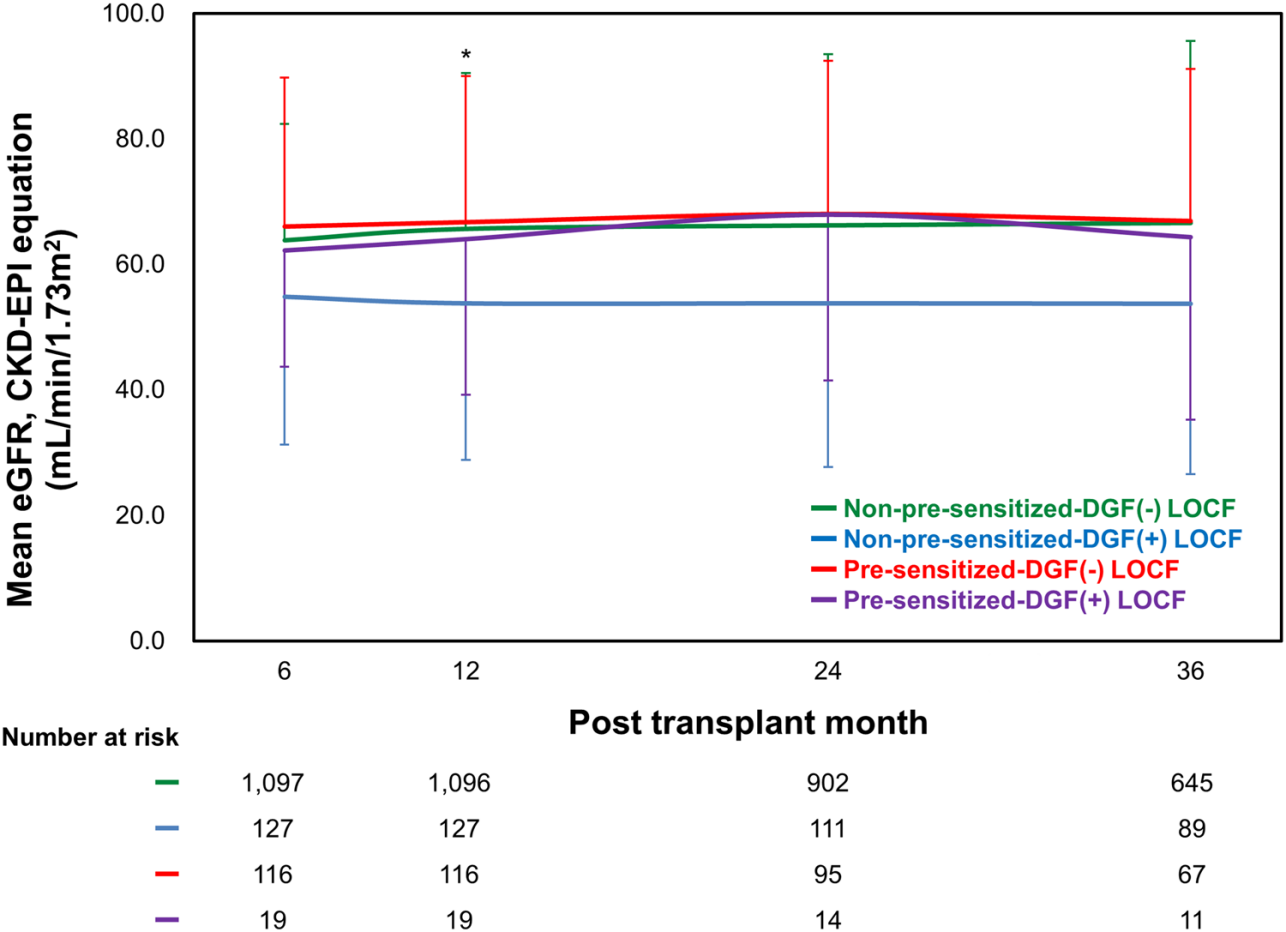
Comparison of the time-related changes in allograft function based on eGFR using CKD-EPI equation (mL/min/1.73 m^2^) according to DGF and pre-sensitization status. During 36 months, the non-pre-sensitized-DGF(+) subgroup showed the lowest allograft function compared with other subgroups. *eGFR* Estimated glomerular filtration rate, *CKD-EPI* chronic kidney disease-epidemiology collaboration, *DGF* delayed graft function. *p < 0.05 non-pre-sensitized-DGF(−) vs. non-pre-sensitized-DGF(+) subgroup.

Since information whether KT recipients were on dialysis at the time of discharge was unavailable, serum creatinine from 6-month after transplantation were used to compare allograft function across 4 subgroups. During 3-year follow-up, allograft function measured by eGFR using chronic kidney disease-epidemiology collaboration (CKD-EPI) equation declined in non-pre-sensitized-DGF(+) subgroup. While the change in time-related allograft function at 12 months from the respective baseline of non-pre-sensitized-DGF(−) subgroup were significantly different from that of non-pre-sensitized-DGF(+) in linear mixed model (p = 0.007), other subgroups showed no significant difference. The change in time-related allograft function at other time points showed no significant difference across 4 subgroups (p = 0.435 at 24 months, p = 0.059 at 36 months) (Fig. 2).

**Figure 3:**
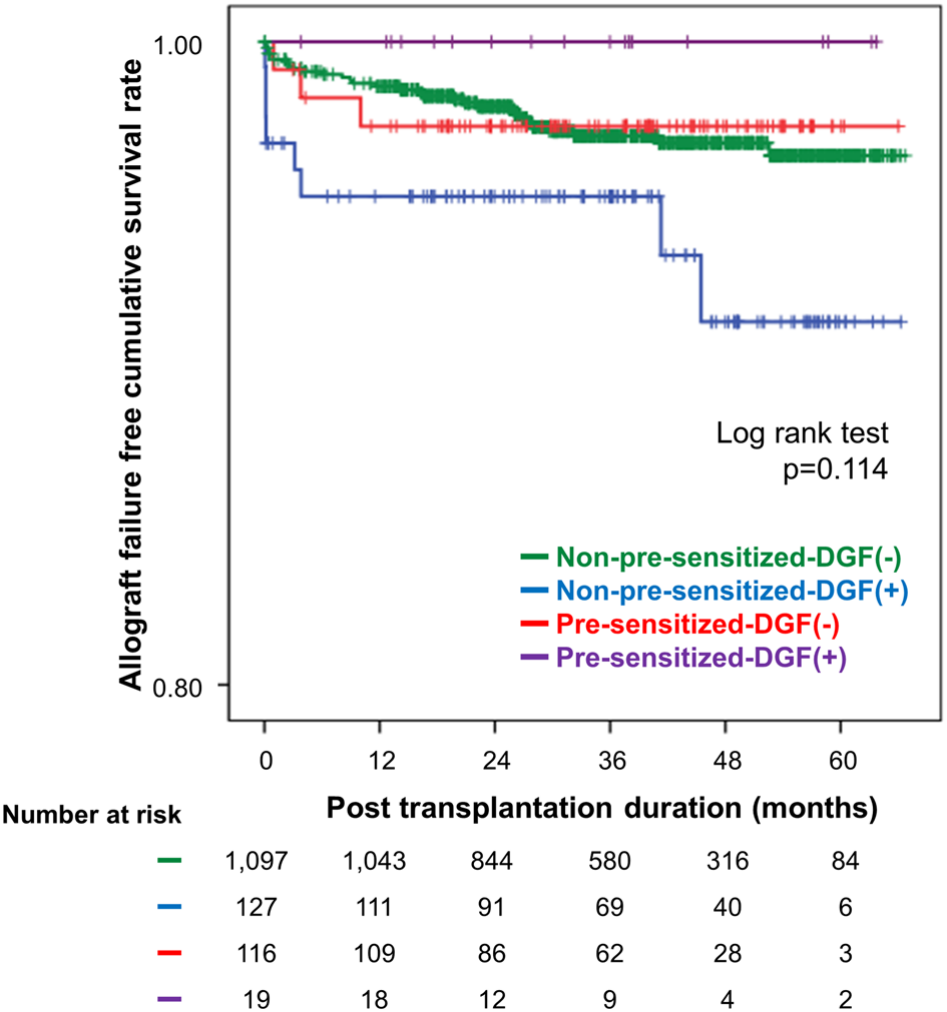
Kaplan–Meier estimates of death-censored allograft survival according to DGF and pre-sensitization status. The numbers below the figures denote the number of KTRs at risk in each subgroup. *DGF* delayed graft function, *KTR* kidney transplantation recipient.

Totally, 41 cases of allograft failure developed during the follow-up period. The median follow-up period of graft failure in each group showed no significant difference [non-pre-sensitized-DGF(−) 37.5 (interquartile range (IQR) 25.0–50.6], non-pre-sensitized-DGF(+) 36.8 (IQR 21.7–58.3), pre-sensitized-DGF(−) 37.7 (IQR 23.6–47.36), and pre-sensitized-DGF(+) 36.0 (IQR 17.7–44.1) months, p = 0.610). The main factor contributing to allograft loss was rejection (15/41, 36.6%). Of these, 5 had clinical rejection, and 10 had BPAR. Acute ABMR occurred in 6/15 (40%), of which 5 were in non-pre-sensitized-DGF(−) subgroup and 1 in pre-sensitized-DGF(−) subgroup. In non-pre-sensitized-DGF(−) subgroup, rejection was the main cause of allograft loss (11/30, 36.7%), followed by unknown (10/30, 33.3%). In non-pre-sensitized-DGF(+) subgroup, the main cause of allograft loss was rejection (3/8, 37.5%). In pre-sensitized-DGF(−) subgroup, both rejection (1/3, 33.3%) and postoperative complications (1/3, 33.3%) accounted for same proportion. In pre-sensitized-DGF(+) subgroup, no allograft loss was reported. The Kaplan–Meier curve showed no significant difference in death-censored allograft survival among 4 subgroups (log rank p = 0.114) (Fig. 3).

### Comparison of patient survival and post-transplant complications

A total of 55 (4.0%) patients died in our cohort due to cardiovascular disease in 9 cases, infection in 26, malignancy in 4, others (liver disease, cerebral infarction, acute CNI toxicity, gastrointestinal bleeding, and acute rejection etc.) in 11, and unknown etiology in 5 cases. In each subgroup, 37 (3.4%) died in the non-pre-sensitized-DGF(−) subgroup, 14 (11.0%) in the pre-sensitized-DGF(+) subgroup, 4 (3.4%) in the pre-sensitized-DGF(−) subgroup, and none (0.0%) in the pre-sensitized-DGF(+) subgroup. The total death rate was the highest in the non-pre-sensitized-DGF(+) subgroup (p = 0.001) (Table 4a).

**Table 4:** (a) Causes of death and (b) clinical outcomes among the 4 subgroups according to DGF and pre-sensitization status.

	Non-pre-sensitized (n = 1224)		Pre-sensitized (n = 135)			*p*-value
	DGF(−) (n = 1097)	DGF(+) (n = 127)	DGF(−) (n = 116)		DGF(+) (n = 19)	
**(a)**						
Total (n, %)	37 (3.4%)	14 (11.0%)	4 (3.4%)		0 (0.0%)	0.001
Cardiovascular disease (n, %)	7 (18.9%)	1 (6.4%)	1 (25.0%)		0 (0.0%)	
Infection (n, %)	20 (54.1%)	4 (31.3%)	2 (50.0%)		0 (0.0%)	
Malignancy (n, %)	1 (2.7%)	2 (12.5%)	1 (25.0%)		0 (0.0%)	
Others (n, %)	8 (21.6%)	3 (25.0%)	0 (0.0%)		0 (0.0%)	
Unknown (n, %)	1 (2.7%)	4 (25.0%)	0 (0.0%)		0 (0.0%)	
**(b)**						
BKVAN (n, %)	32 (2.9%)	4 (3.1%)	5 (4.3%)		0 (0.0%)	0.729
Cardiovascular disease (n, %)	141 (12.9%)	24 (18.9%)	14 (12.1%)		0 (0.0%)^†^	0.081
Cerebrovascular disease (n, %)	10 (0.9%)	1 (0.8%)	0 (0.0%)		0 (0.0%)	0.740
**Infection**						
Overall (n, %)	273 (32.9%)	37 (39.4%)	37 (42.0%)		3 (23.1%)	0.185
CMV infection (n, %)	49 (5.9%)	4 (4.3%)	1 (1.1%)		0 (0.0%)	0.203
PJP infection (n, %)	5 (0.6%)	1 (1.1%)	1 (1.1%)		0 (0.0%)	0.889
Malignancy (n, %)	77 (7.0%)	11 (8.7%)	9 (7.8%)		2 (10.5%)	0.848

Continuous variables are shown as mean ± standard deviation or median with interquartile range. Categorical variables are shown as number (proportions).

Others: liver disease, cerebral infarction, acute CNI toxicity, gastrointestinal bleeding, intestinal obstruction, acute rejection etc.

*DGF* delayed graft function, *CNI* calcineurin inhibitor, *BKVAN* BK virus associated nephropathy, *CMV* cytomegalovirus, *PJP* *Pneumocystis jirovecii* pneumonia.

There was no significant difference in development of BK virus-associated nephropathy (BKVAN), cerebrovascular disease, infectious complications and malignancy across the 4 subgroups (Table 4b).

## Discussion

Pre-sensitization to HLA is a well-known pre-transplant factor, which can increase the risk for allograft rejection and allograft failure. Meanwhile, DGF is a well-known post-transplant factor, which also induces adverse allograft outcomes. This study demonstrated that the combination of post-transplant factor (DGF) and pre-transplant risk factor (pre-sensitization) had a synergistic adverse effect on allograft outcomes, especially higher incidence of allograft rejection.

First, we compared baseline characteristics of donors and recipients across 4 clinical subgroups. In terms of donor factors, baseline renal function was significantly lower in patients who showed DGF, which was consistent with previous studies, which reported that low baseline kidney function is a risk factor for DGF^13^. In contrast, there was no significant difference in the frequency of DGF between pre-sensitized and non-pre-sensitized subgroups, which suggests that pre-sensitization may not have a significant effect on the development of DGF. Among recipient factors, the dialysis was significantly prolonged in pre-sensitized subgroups, which suggested that sensitized subjects need longer wait time for DDKT allocation^14–16^. As expected, the proportion of female recipients was higher in both pre-sensitized subgroups^15^ and the proportion of recipients with previous KT history was higher and tended to be high in both pre-sensitized subgroups than in non-pre-sensitized subgroups. In addition, although a majority of patients received primary maintenance immunosuppression with tacrolimus, a higher number of patients tended to receive sirolimus in the non-pre-sensitized-DGF(+) subgroup. This finding suggested that physicians decided a switch from CNI to mammalian target of rapamycin (mTOR) inhibitor, given that CNI may contribute to delayed recovery of allograft function^17^.

Second, we compared the incidence of overall BPAR according to pre-sensitization or the development of DGF. As a result, the incidence of overall BPAR showed a tendency to be higher in the pre-sensitized-DGF(+) subgroup, and that of acute ABMR was the highest in the pre-sensitized-DGF(+) subgroup. Interestingly, pre-sensitization and DGF showed significant interaction with each other, which suggests their synergistic impact on the development of overall BPAR and acute ABMR. This finding can be explained by two factors. First, DGF per se can increase the immunogenicity of allograft, and thereby increase the vulnerability to immune reaction of pre-formed HLA-DSA. Indeed, IRI in DGF can up-regulate the major histocompatibility complex (MHC) class I and II antigens, and enhance the expression of adhesion and costimulatory molecules of allograft tissue^18–21^. Moreover, the IRI induces ligands of toll-like receptors (TLRs) and activate cells of the innate immune system, inducing activation and maturation of dendritic cells, followed by adaptive immune response^21^. Indeed, the previous studies demonstrated that DGF is associated with an increased risk of allograft loss and acute rejection^22,23^. Second, the conversion of CNI to mTOR inhibitor was more frequently detected in patients who suffered from DGF in this study, perhaps because CNI might be considered as a contributor to DGF. Lower suppressive potency of mTOR inhibitor for humoral immunity in comparison with tacrolimus is another possible cause of higher rate of acute ABMR in pre-sensitized-DGF(+) subgroup^24^.

Surprisingly, pre-sensitization or DGF per se had no significant effect on the development of overall BPAR. The reason is unclear, but it may be attributed to the limited definition of both pre-sensitization and DGF in the study using a nationwide cohort, retrospectively. In case of pre-sensitization, since data of DSA were collected from 2017, the results of HLA-DSA were not available in some recipients. Therefore, in such recipients, we defined sensitization to HLA by the presence of panel reactive antibodies (PRA), together with positive crossmatch test results. Even though this definition is used for “pre-sensitization”, we cannot assess the degree of sensitization clearly. In case of DGF, the definition of DGF is varies among previous studies^25^. Indeed, the definition of DGF merely depends on the performance of dialysis after KT, and the decision whether or not to perform dialysis can differ according to the transplant centers. In addition, due to the absence of detail data, individualized immunosuppression regimen according to immunologic risk stratification and the serum level of immunosuppressant in each recipient did not be considered in our analysis. Therefore, the aforementioned factors can induce bias that can affect the result of this study.

Interestingly, non-pre-sensitized-DGF(+) group showed worst allograft function at 36 months post-transplant follow-up. One of the possible reasons is the baseline status of the corresponding donor kidney (Supplementary Table 1). The donor of this group showed relatively lower renal function at baseline, longer cold ischemic time, and a higher kidney donor profile index (KDPI) score, even though statistically insignificant. All of the foregoing findings suggest that the baseline status of donor kidney was the worst in this group, which may result in sustained low allograft function. In regard to allograft function, the impact of the baseline kidney function can be more significant than allograft rejection during the limited follow-up duration. Hence, the allograft survival was not different across 4 subgroups.

Lastly, we compared the post-transplant complications among the 4 subgroups. Non-pre-sensitized-DGF(+) subgroup showed the higher patient death rate. However, only 55 cases out of 1359 KTRs were found and there was no patient death in the pre-sensitized-DGF(+) subgroup. Therefore, longer observations may be required to arrive at any conclusion. Compared with post-transplant complications, no difference was detected across 4 subgroups in the development of BKVAN, cardiovascular disease, cerebrovascular disease, infection, and malignancy. However, further investigation may be required to clarify this issue^26^.

This study has some limitations. First, this nationwide registry analysis reflects similar limitations found in similar large registry analyses as shown in our previous studies^27^. While patient numbers are enhanced, important details for the endpoints are missing, thereby reducing the clinical utility of the findings. For example, the HLA-DSA was not available for analysis in some patients (22.6%). Additionally, MFI cut-off to define positive at respective centers was not available, and we cannot use the class and the strength of DSA in the analysis, which has been reported as an important risk factor for allograft rejection and failure^6,10,28–30^. Second, the follow-up duration of this registry is limited as mentioned previously. Therefore, traditional risk factors for allograft failure such as DGF and pre-sensitization did not significantly affect allograft outcome. Third, we could not determine the specified protocols at each center in DDKT for highly sensitized recipients, such as desensitization and surveillance biopsy protocols. Despite pre-transplant desensitization was performed in 35 recipients, including those whom with positive B-cell crossmatch, no data were available on the protocol. Some centers used rituximab to treat such patients, and others did not, but unfortunately, it was not considered in this analysis. Nevertheless, our study is the first multi-centered cohort study to investigate the association of DGF and pre-sensitization in allograft outcomes.

In conclusion, we have shown that combination of DGF and pre-sensitization to HLA had a detrimental impact on allograft outcome in terms of rejection. Therefore, we suggest that more careful monitoring or surveillance of allograft rejection is required. Further, we need to use more intensified immunosuppression protocol to prevent allograft rejection when DGF occurred in DDKT with pre-sensitization.

## Methods

### Study population

**Figure 4:**
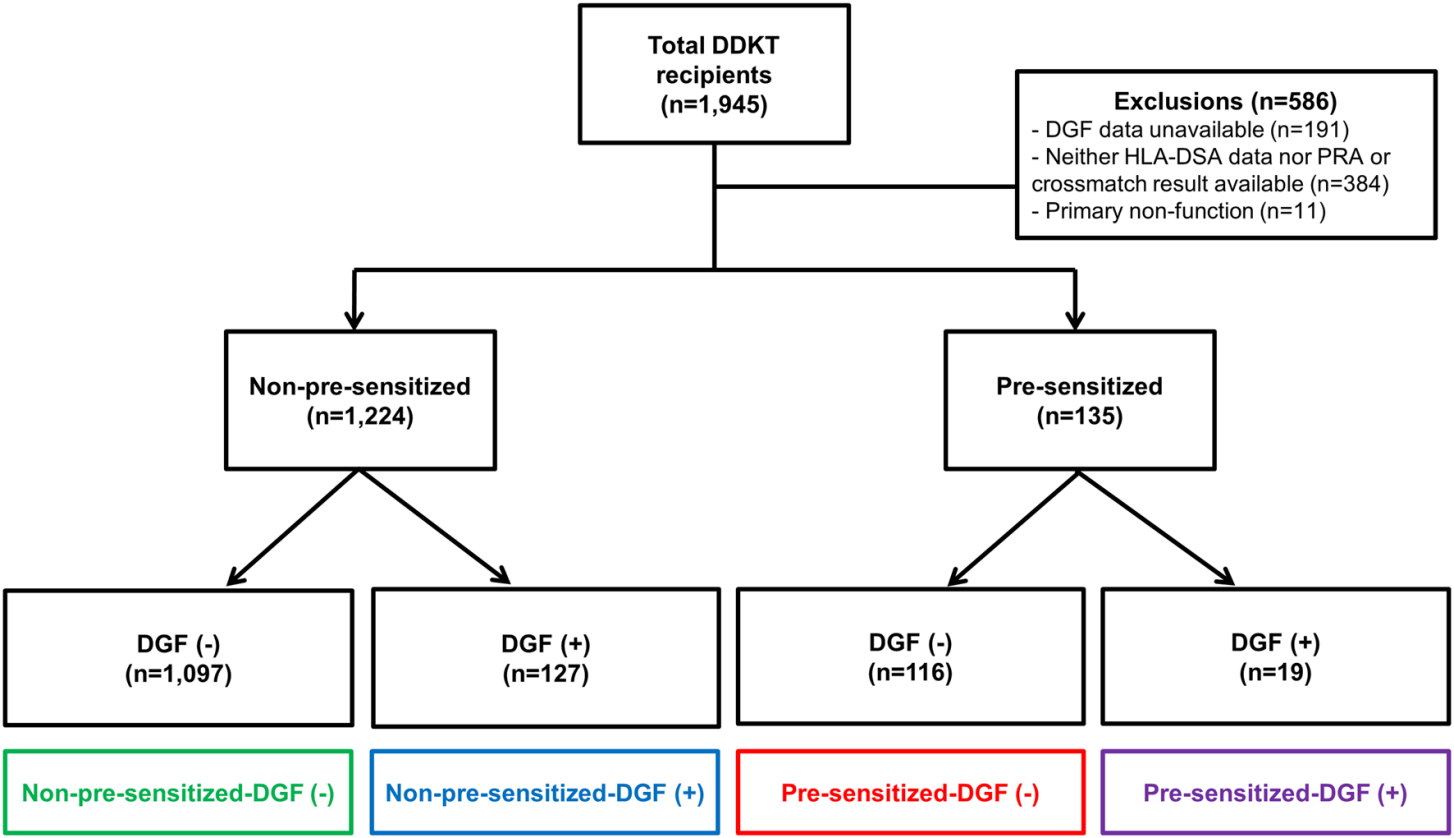
Distribution of the patient population according to DGF or pre-sensitization to HLA. *DGF* delayed graft function, *HLA* human leukocyte antigen, *DDKT* deceased donor kidney transplantation, *DSA* donor-specific antibody.

We analysed KOTRY data from the Korean Society for Transplantation^31^, compiling data from 30 kidney transplantation centers in Korea^32^. The KOTRY data includes 1945 DDKT cases between May 2014 and June 2019, from which we excluded 586 DDKT recipients with unavailable data regarding PRA, HLA-DSA, crossmatch tests or DGF development, and with primary non-function of the kidney allograft. Therefore, we included 1359 DDKT recipients in the present investigation and classified the patients into four subgroups according to the pre-sensitization and the development of DGF post-transplant: non-pre-sensitized-DGF(−) (n = 1097), non-pre-sensitized-DGF(+) (n = 127), pre-sensitized-DGF(−) (n = 116), pre-sensitized-DGF(+) (n = 19) (Fig. 4). The median follow-up period of this study was 38.1 (IQR 25.2–50.8) months.

We defined pre-sensitization to HLA by the presence of (i) HLA-DSA (by Luminex single antigen assay) or (ii) PRA (by solid-phase HLA antibody screening), combined with positive crossmatch test results. HLA-DSA data were available in 1052 recipients (77.4%). Therefore, the sensitization to HLA was defined by the detection of HLA-DSA in those patients. In another 307 (22.6%) DDKT recipients for whom HLA-DSA data were not available, we defined sensitization to HLA based on the positive results of PRA and crossmatch test, regardless of whether complement-dependent cytotoxicity or flow cytometry. DGF was defined as the need for dialysis within 1 week of transplantation. The medical records were reviewed after receiving informed consent^32^. This study was performed in accordance with the Declaration of Helsinki and the Declaration of Istanbul. The study was approved by the Institutional Review Board of Seoul St. Mary’s Hospital (KC14ONMI0460).

### Definition of clinical outcomes

The clinical outcomes investigated in this study included the incidence of overall BPAR, acute ABMR, time-related changes in allograft function measured as eGFR, death-censored allograft survival rates, and post-transplant complications such as BKVAN, cardiovascular disease, cerebrovascular disease, infection and malignancy. BPAR was diagnosed according to the Banff 2013 classification^33^. Rejection-free allograft survival was defined as the time elapsed from transplantation to the first episode of BPAR. Serum creatinine levels were collected at six months and later at one-year intervals post-transplant. The eGFR for each concordant time was assessed using the CKD-EPI equation^34^. Allograft survival was defined as the time from transplantation to initiation with alternative renal replacement therapy. Cardiovascular disease is defined as cardiovascular death, myocardial infarction, ischemic heart disease with relevant clinical evidence (accompanied by therapeutic intervention or objective findings), new-onset congestive heart failure requiring hospitalization, and arrhythmia. Cerebrovascular disease included non-traumatic hemorrhagic or ischemic brain disease confirmed by computed tomography or magnetic resonance imaging^32^. BKVAN was diagnosed by allograft biopsy. All clinical parameters were compared across the four patient subgroups.

### Statistical method

All continuous variables were expressed as mean ± standard deviation. If the variables followed the normal distribution, an analysis of variance (ANOVA) was performed. If the variables showed non-normal distribution, a Kruskal–Wallis test was performed. Tukey’s method or Mann–Whitney test was performed as a post-hoc analysis. All categorical variables were compared using the chi-square test or Fisher's exact test and expressed as proportions. Withdrawal-censored allograft rejection rate and death-censored allograft survival rate were evaluated by using the Kaplan–Meier survival analysis and were compared using the log-rank test. The effects of DGF and pre-sensitization, and the interaction between DGF and pre-sensitization on overall BPAR or acute ABMR were analyzed via Cox proportional-hazards regression analysis. Baseline clinical and laboratory parameters showing significant differences (p value < 0.05) in univariable analysis or known to affect allograft rejection were fitted into the multivariable model. We selected donor factors (cold ischemic time, KDPI) and recipient factors (BMI, dialysis duration, mismatch number, previous KT history, PRA > 50%) as confounders. Time-related allograft function between subgroups were compared using a linear mixed model. The last observation carried forward (LOCF) analysis was used for missing eGFR values. All missing data were censored from the last follow-up date. p values < 0.05 were statistically significant. All statistical analyses were performed using the SPSS^®^ software, version 24 (IBM Corporation, Armonk, NY, USA) and Microsoft Excel 2016.

## Supplementary Information

Supplementary Table 1.

## Data Availability

The datasets generated during and/or analysed during the current study are available from the corresponding author on reasonable request.
