# Supplementary material for: Synergistic impact of pre-sensitization and delayed graft function on allograft rejection in deceased donor kidney transplantation
Source: Sci Rep. 2021 Aug 9;11:16095. doi: 10.1038/s41598-021-95327-6 (PMC8352860; doi:10.1038/s41598-021-95327-6)
Supplement: Supplementary file 1 — Supplementary Table 1. [file 41598_2021_95327_MOESM1_ESM.doc]

# Synergistic Impact of Pre-sensitization and Delayed Graft Function on Allograft Rejection in Deceased Donor Kidney Transplantation

Hanbi Lee^1,+^, Yohan Park^1, +^, Tae Hyun Ban^2^, Sang Heon Song^3^, Seung Hwan Song^4^, Jaeseok Yang^5^, Curie Ahn^5^, Chul Woo Yang^1^, Byung Ha Chung^1,*^ & the Korean Organ Transplantation Registry Study Group**^‡^**

^1^ Division of Nephrology, Department of Internal Medicine, Seoul St. Mary’s Hospital. ^2^ Division of Nephrology, Department of Internal Medicine, Eunpyeong St. Mary’s Hospital. ^3^ Organ Transplantation Center and Department of Internal Medicine, Pusan National University Hospital. ^4^ Department of Surgery, Ewha Womans University Medical Center. ^5^ Department of Nephrology, Seoul National University Hospital.

^+^ These authors contributed equally to this work.

^‡^ A comprehensive list of the Korean Organ Transplantation Registry Study Group members appears at the end of the paper.

^*^ email: [**chungbh@catholic.ac.kr**](mailto:chungbh@catholic.ac.kr)

Yohan Park's current affiliation is ‘Division of Nephrology, Department of Internal Medicine, Konyang University Hospital, College of Medicine, Konyang University, Daejeon, Republic of Korea.’.

**Supplemental Information**

**Table S1. Comparison of baseline characteristics of donors according to allograft failure.**

|  | Allograft loss (-)  (n = 1,318) | Allograft loss (+)  (n = 41) | *p*-value |
| --- | --- | --- | --- |
|  |
| Age (years) | 47.8 ± 14.7 | 48.8 ± 15.1 | 0.667 |
| KDPI | 66.0 (47.0-84.0) | 71.0 (41.0-82.0) | 0.550 |
| HTN (n, %) | 309 (24.9%) | 11 (29.7%) | 0.563 |
| DM (n, %) | 144 (11.5%) | 2 (5.1%) | 0.305 |
| Cold ischemic time (min) | 267.5 (204.0-358.0) | 310.0 (200.0-420.0) | 0.220 |
| eGFR (CKD-EPI) (ml/min/1.73m^2^) | 70.6 (38.3-105.2) | 69.7 (37.2-107.7) | 0.476 |

Continuous variables are shown as mean ± standard deviation or median with interquartile range. Categorical variables are shown as number (proportions). HTN, hypertension; DM, diabetes mellitus; eGFR, estimated glomerular filtration rate; CKD-EPI, chronic kidney disease-epidemiology collaboration**.**
